# Russet Susceptibility in Apple Is Associated with Skin Cells that Are Larger, More Variable in Size, and of Reduced Fracture Strain

**DOI:** 10.3390/plants9091118

**Published:** 2020-08-29

**Authors:** Bishnu P. Khanal, Thi Lieu Le, Yiru Si, Moritz Knoche

**Affiliations:** Institute for Horticultural Production Systems, Leibniz University Hannover, 30419 Hannover, Germany; lelieu81vn@gmail.com (T.L.L.); yiru.si@obst.uni-hannover.de (Y.S.); moritz.knoche@obst.uni-hannover.de (M.K.)

**Keywords:** *Malus × domestica*, russet, epidermis, hypodermis, mechanical properties

## Abstract

Russeting is an economically important surface disorder in apple (*Malus × domestica* Borkh). Indirect evidence suggests an irregular skin structure may be the cause of the phenomenon. The objective of this study was to characterize epidermal and hypodermal cell morphology and the mechanical properties of the skins of apple cultivars of differing russet susceptibility. Dimensions of epidermal and hypodermal cells were determined using microscopy. Stiffness (*S*), maximum force ($F_{max}$), and maximum strain ($\varepsilon_{max}$) at failure were quantified using uniaxial tensile tests of skin strips. Particularly during early fruit development, epidermal cells (EC) and hypodermal cells (HC) in russet non-susceptible cultivars occurred in greater numbers per unit area than in russet-susceptible ones. The EC and HC were lower in height, shorter in length, and of reduced tangential surface area. There were little differences in *S* or $F_{max}$ between non-susceptible and susceptible cultivars. However, the $\varepsilon_{max}$ were higher for the skins of non-susceptible cultivars, than for those of susceptible ones. This difference was larger for the young than for the later growth stages. It is concluded that russet-susceptible cultivars generally have larger cells and a wider distribution of cell sizes for both EC and HC. These result in decreased $\varepsilon_{max}$ for the skin during early fruit development when russet susceptibility is high. This increases the chances of skin failures which is known to trigger russeting.

## 1. Introduction

Russeting is an economically important surface disorder in many fruitcrop species including in apple (*Malus × domestica* Borkh.) [1]. Russet spoils the smooth, shiny appearance of the fruit skin but does not affect its nutritional value. Thus, even though the effect of russet is merely cosmetic, for smooth-skinned apple cultivars it results in a downgrading of the fruit and so can be a cause of serious financial loss for the fruit grower. In other apple cultivars (e.g., “Cox’s Orange Pippin”), a small amount of russet around the stalk cavity is accepted as a characteristic of that cultivar. 

In botanical terms russet represents a periderm comprising a phellogen that divides and generates layers of cork cells, the phellem. The cell walls of the phellem become impregnated with suberin resulting in the typical brownish, dull appearance of a russeted fruit skin. Russet begins with the formation of microcracks in the cuticle. These usually occur above, and are aligned with, the anticlinal cell walls of the epidermis [1,2,3,4]. Microcracks impair the barrier properties of the cuticle [2,5]. Initially, microcracks are shallow and do not fully traverse the thickness of the cuticle, possibly due to a gradient in strain across the cuticle [6]. Shallow microcracks may be repaired by subsequent depositions of wax [2,7]. 

More severe microcracks traverse the cuticle and extend into the epidermal cell layer and below. It is this deeper type of microcracking that triggers the development of a periderm [3,8]. The periderm begins to form in the hypodermis [8]. The fruit’s primary skin (the epidermis) dries. It is subsequently shed; thus, exposing the periderm at the fruit surface. This appears as a non-shiny, rough, brownish, corky layer [1,8]. This sequence of changes is referred to as russeting. Apple fruit are particularly susceptible to russet during the early stages of development [1,3,9,10,11,12]. 

Apple cultivars differ in their susceptibility to russeting [13]. Based on its susceptibility to russeting, a cultivar can be assigned to one of three arbitrary susceptibility categories: low, intermediate, or high. When grown under the same "average" environmental conditions, apple cultivars of the first category will not russet, those of the second may develop some russet and those of the third will always develop russet (Khanal’s personal observation). Cultural factors also have an effect. Thus, extended periods of surface wetness and/or exposure to high humidity are key factors inducing russet in the susceptible cultivars [3,11,12,14,15,16]. Meanwhile, the application of gibberellins A_4 + 7_ (GA_4 + 7_) can reduce the incidence of russeting [12,17,18,19,20,21]. 

The factors responsible for the differences in russet susceptibility among apple cultivars and between treatments and due to surface wetness/humidity etc., are unknown. An earlier study found no relationship between the physical and mechanical properties of the cuticle at maturity and the susceptibility to russet among 22 different apple cultivars [13]. The decreased russet susceptibility of GA-treated apples was attributed to an increase in cell density (more cells per unit surface area and hence, smaller cell size) in the epidermis [17]. Application of GA had no effect on cuticle mass per unit area or on the mechanical characteristics of cuticles [12]. These findings are consistent with the observation that the rheological properties of the apple fruit skin are principally determined by the epidermal and hypodermal cell layers, and not by the cuticle [22]. Furthermore, even the pattern formed by the microcracks in the cuticle is accounted for by the disposition of the anticlinal cell walls of the underlying epidermis [2]. All these findings indicate the factor(s) determining russet susceptibility (1) more likely reside with the cellular components of the fruit skin, rather than with the cuticle, and (2) that they are especially relevant during the early stages of fruit development, when susceptibility to russet is highest.

The objective of this study was to test these two hypotheses. We focused (1) on the morphology of the epidermal and hypodermal cells and (2) on the mechanical properties of the skins of two representative apple cultivars—one cultivar that is particularly russet-susceptible (“Karmijn”) and one that is particularly russet non-susceptible (“Idared”). We recorded changes in the mechanical properties of these two cultivars during fruit development and identified those properties that differed most. We then examined the same properties in seven other apple cultivars of contrasting russet susceptibility—four known to be especially russet-susceptible and three known to be especially russet non-susceptible.

## 2. Results

### 2.1. Morphology of the Skin of Developing Karmijn and Idared Apple

**Figure 1:**
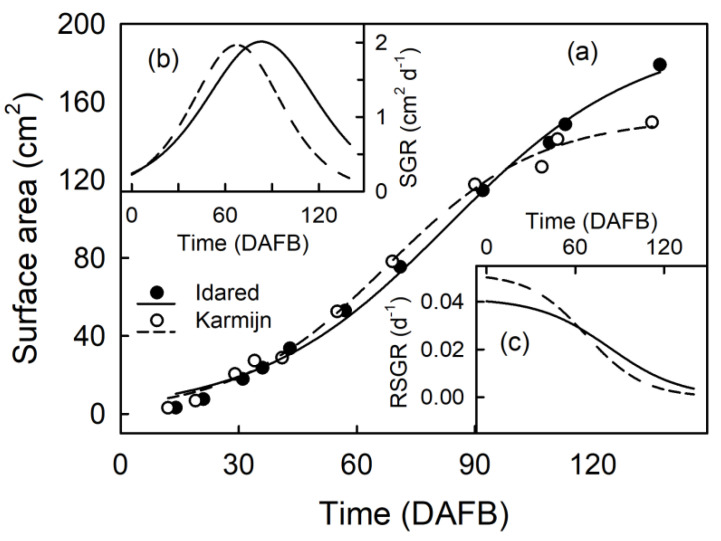
Time courses of change in fruit surface area of developing ‘Karmijn’ and ‘Idared’ apples (**a**). Fruit surface area growth rate (SGR; **b**) and relative fruit surface area growth rate (RSGR; **c**) as affected by time. Data represent means ± SE. The minimum number of replicates was 40 fruit. Error bars are not visible, as they are smaller than the data symbols. DAFB = days after full bloom.

The surface areas of the Karmijn and Idared apples increased with time in a sigmoidal pattern (Figure 1a). The maximum surface area growth rates (SGR) were similar in the two cultivars. Rates peaked at 65 in Karmijn and at 80 days after full bloom (DAFB) in Idared (Figure 1b). The maximum relative surface area growth rate (RSGR) was highest early on and then decreased. The RSGR was higher in Karmijn than in Idared apple (Figure 1c). 

**Figure 2:**
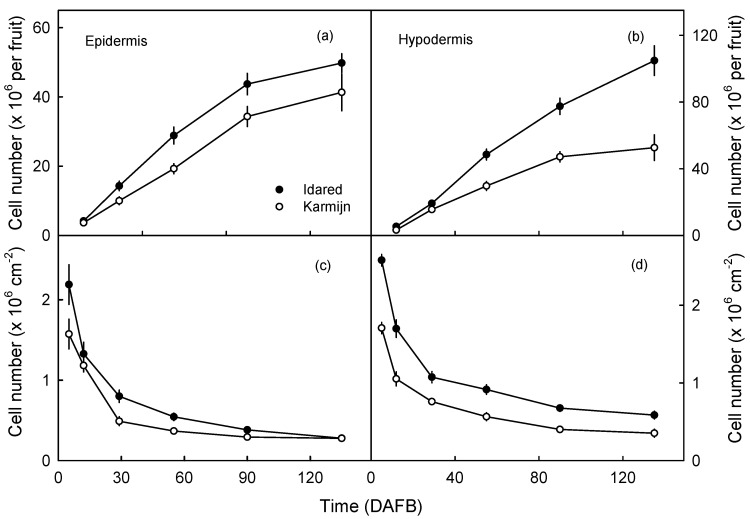
The time courses of change in numbers of epidermal (**a**,**c**) and hypodermal (**b**,**d**) cells per fruit (**a**,**b**) and per unit fruit surface area (**c**,**d**) in ‘Karmijn’ and in ‘Idared’ apples. Data represent means ± SE. The number of replicates was seven. Where error bars are not visible, they are smaller than the data symbol. DAFB = days after full bloom.

The number of epidermal cells (EC) and hypodermal cells (HC) per fruit increased with time but at a decreasing rate. Both cell numbers were higher in Idared than in Karmijn (Figure 2a,b). At the same time, the numbers of EC and HC per unit surface area decreased. Numbers were always higher in Idared than in Karmijn (Figure 2c,d).

**Figure 3:**
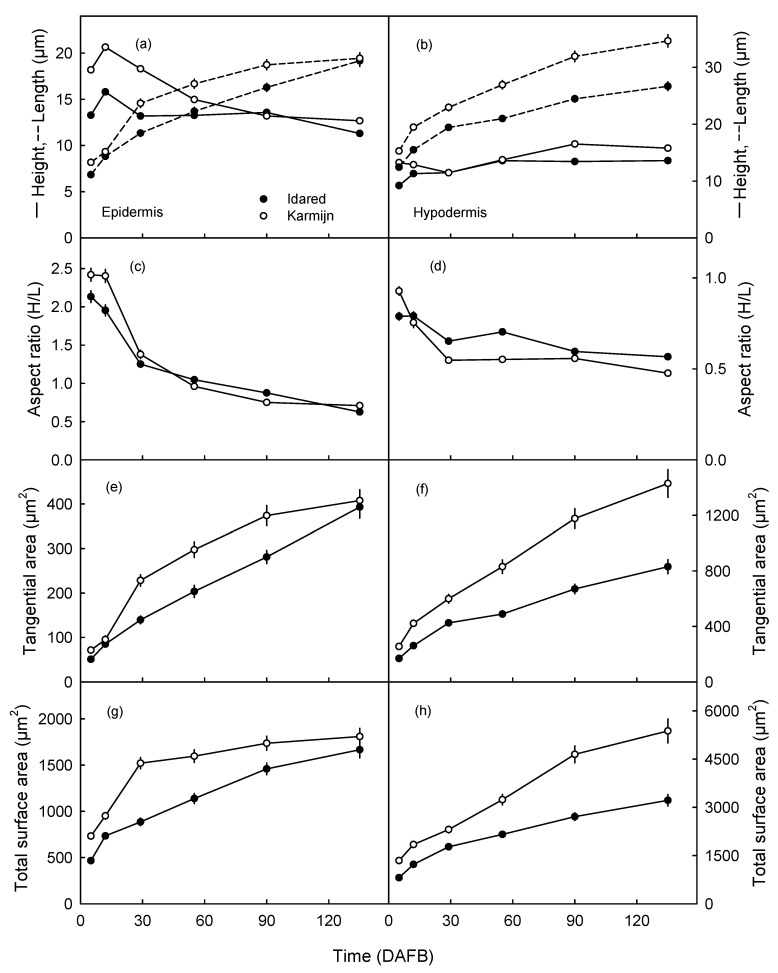
Time courses of change in height and length (**a**,**b**), aspect ratio (height/length; **c**,**d**), tangential area (**e**,**f**) and total surface area (**g**,**h**) of epidermal (**a**,**c**,**e**,**g**) and hypodermal (**b**,**d**,**f**,**h**) cells of ‘Idared’ and ‘Karmijn’ apples. Cell surface area was calculated from cell height and length assuming cells were rectangular prisms. For detail see Methods. Data represent means ± SE. The numbers of replicates were 68–100 (epidermis) and 179–228 (hypodermis) cells. Cells were measured in seven fruit. Where error bars are not visible, they are smaller than the data symbol. DAFB = days after full bloom.

The lengths of the EC and HC increased continuously from 5 to about 90 DAFB in both cultivars (Figure 3a,b). The initial length and the rate of increase in length during the early stages of fruit development were larger in Karmijn than in Idared apple. In the epidermis, the length difference between the two cultivars disappeared as the fruit approached maturity (Figure 3a) but in the hypodermis, cell length in Karmijn consistently exceeded that in Idared (Figure 3b). 

The EC were initially higher in Karmijn than in Idared. During development, EC height decreased slightly in both cultivars. The height difference eventually disappeared with later fruit development (Figure 3a). The height of the HC remained constant during development and was similar for Karmijn and Idared (Figure 3b). The size of the HC increased consistently from the outer layer below the epidermis towards the inner layer of the hypodermis adjacent to the parenchyma.

The aspect ratios of the EC and the HC decreased during development in both Karmijn and Idared. Aspect ratios of the EC and HC were also similar in the two cultivars throughout fruit development (Figure 3c,d).

The tangential area (Figure 3e) and total surface area (Figure 3g) of the EC increased asymptotically. These rates of area increase were higher in Karmijn than in Idared, particularly during the early stages of fruit development. The differences decreased with development and essentially disappeared as the fruit matured. Both the HC tangential area (Figure 3f) and total surface area (Figure 3h) increased throughout development. The rates of this increase were higher in Karmijn than in Idared throughout development.

**Figure 4:**
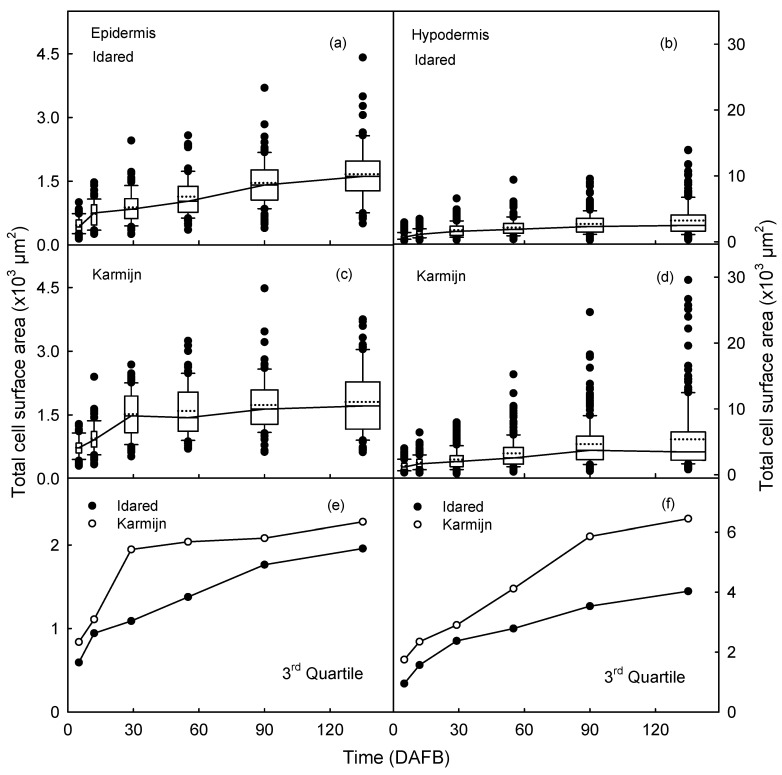
Boxplots of total surface area of epidermal (**a**,**c**) and hypodermal (**b**,**d**) cells at various stages of fruit development of ‘Idared’ (**a**,**b**) and ‘Karmijn’ (**c**,**d**) apples. The 3rd quartile values of the total surface area of epidermal (**e**) and hypodermal (**f**) cells. The numbers of replicates were 68–100 (epidermis) and 179–228 (hypodermis) cells. Cells were measured in seven fruit.

Boxplots of total surface area of the EC revealed a wider distribution in Karmijn than in Idared, at most stages of development (Figure 4a,c). In Idared, the distribution of total surface area of HC was narrower than that of the EC or compared to the HC in Karmijn (Figure 4b,d). Calculating the third quartile and plotting against time revealed consistently higher 3rd quartiles for Karmijn compared to Idared for both the EC and the HC (Figure 4e,f).

### 2.2. Morphology of the Skin of Susceptible and Non-Susceptible Cultivars 

The surface area of young fruit of susceptible cultivars was higher than that of the non-susceptible cultivars. At maturity, the surface area of non-susceptible cultivars was generally higher than that of the susceptible cultivars (Table 1). Additionally, during the early stage of development, the non-susceptible cultivars had significantly greater EC numbers per unit surface area than the susceptible cultivars (Table 1). This difference essentially disappeared in mature fruit. The HC numbers per unit surface area were also higher in the non-susceptible than the susceptible cultivars both in young and in mature stages (Table 1).

The numbers of EC and HC per fruit were the same in both groups of cultivars in young fruit but were higher in the non-susceptible than in the susceptible cultivars at maturity (Table 1).

In young fruit, the heights and lengths of the EC and HC were smaller in the non-susceptible than in the susceptible cultivars (Table 2). The difference in the length of the HC, but not of the EC, continued through to maturity. Accordingly, the tangential surface areas and total surface areas of the EC and HC were smaller in young fruit of the non-susceptible cultivars than of the susceptible ones. For the HC, but not for the EC, this difference persisted till maturity (Table 2).

**Figure 5:**
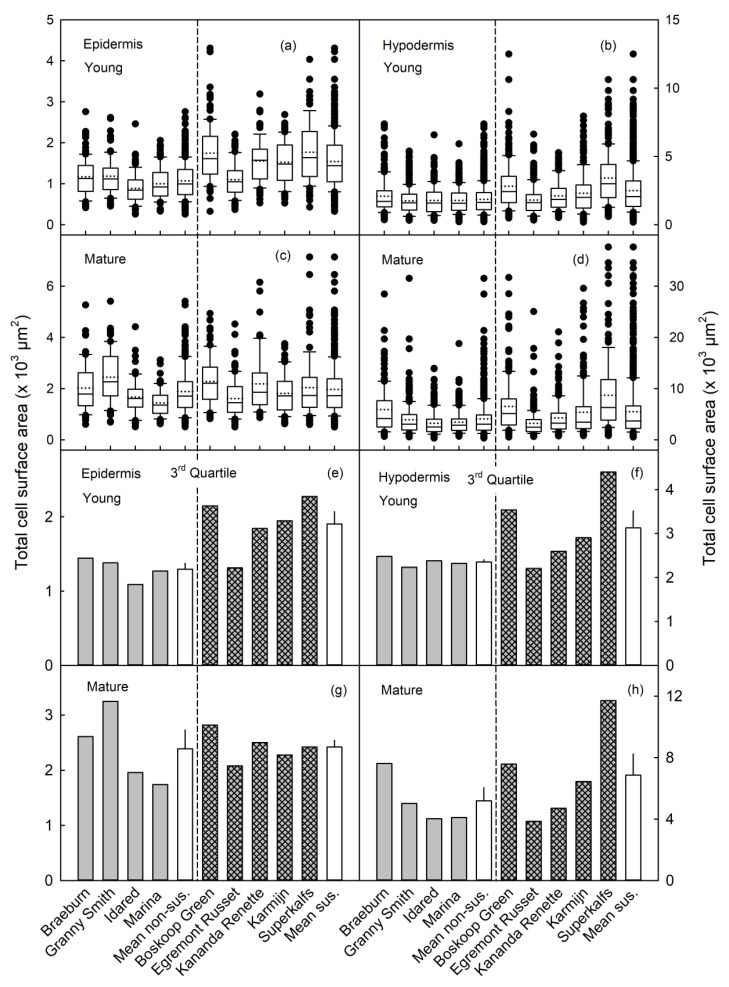
Boxplots (**a**–**d**) of total surface area of epidermal (**a**,**c**) and hypodermal (**b**,**d**) cells of young (28–35 days after full bloom) (**a**,**b**) and mature (**c**,**d**) fruit of selected russet non-susceptible (**left**) and susceptible (**right**) groups of apple cultivars (separated by a vertical dashed line). Values of the 3rd quartile of the total surface area (**e**–**h**) of epidermal (**e**,**g**) and hypodermal (**f**,**h**) cells of young (**e**,**f**) and mature (**g**,**h**) fruit of selected russet non-susceptible (**left**) and susceptible (**right**) groups of apple cultivars (separated by a vertical dashed line). The grand mean for the russet non-susceptible group is referred to as "Mean non-sus.", and that of the russet susceptible group referred to as "Mean sus.". Results for ‘Idared’ and ‘Karmijn’ were taken from Figure 3. The numbers of replicates were 68–145 (epidermis) and 172–331 (hypodermis) cells in young and 57–97 (epidermis) and 128–205 (hypodermis) cells in mature fruit. Cells were measured in seven fruit per cultivar.

A closer look at the distribution of the total cell surface area of the EC and HC reveals a broader distribution for the young fruit of three of the five susceptible cultivars, when compared to that of the non-susceptible cultivars in young fruit (Figure 5). This pattern was also evident in the cell-size data that were pooled within the two groups of cultivars. Again, the broader distributions were obtained in the susceptible rather than in the non-susceptible cultivars (Figure 5a,b). "Egremont Russet" and "Kanada Renette" (both susceptible) were somewhat exceptional, their cell size distributions were more similar to those of the non-susceptible cultivars (Figure 5a,b). 

There were no consistent differences in the distributions of EC size or of HC size in mature fruit between the russet susceptible and non-susceptible cultivars (Figure 5c,d).

The value of the third quartile of the frequency distribution of total surface area of EC and HC was higher in most russet susceptible cultivars than in non-susceptible cultivars in young fruit. The third quartile value of EC and HC of the pooled data of susceptible cultivars was also higher than that of the non-susceptible cultivars (Figure 5e,f). There were no consistent differences in the third quartile values of the EC and HC size distributions between the susceptible and non-susceptible groups of cultivars at maturity (Figure 5g,h).

### 2.3. Skin Rheology of Developing Karmijn and Idared Apples 

**Figure 6:**
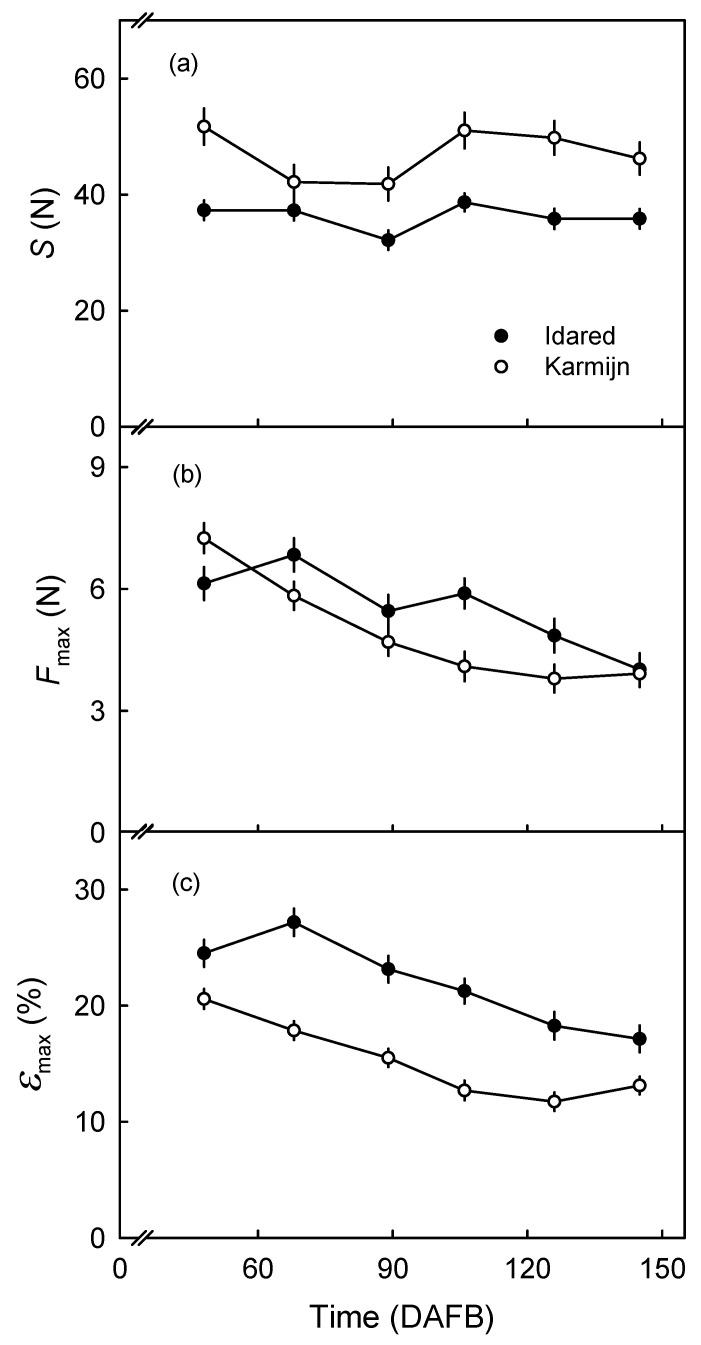
Developmental time courses of change in stiffness (*S*; **a**), maximum force ($F_{max}$; **b**) and maximum strain ($\varepsilon_{max}$; **c**) of exocarp segments (ES) of 0.5 mm standard thickness, excised from the equatorial surface of ‘Karmijn’ and ‘Idared’ apples. The number of replicates was 25–35.

Uniaxial tensile tests revealed that the *S* of the exocarp segments (ES) remained constant during development being generally larger for Karmijn than for Idared (Figure 6a). The $F_{max}$ and $\varepsilon_{max}$ both decreased continuously with development in both cultivars. With few exceptions, both values were lower in Karmijn than in Idared (Figure 6b,c). The exceptions were the first sampling at 49 DAFB where $F_{max}$ of Karmijn exceeded that of Idared and at maturity where the $F_{max}$ of both cultivars were the same (Figure 6b).

### 2.4. Rheology of the Skin of Susceptible and Non-Susceptible Cultivars

**Figure 7:**
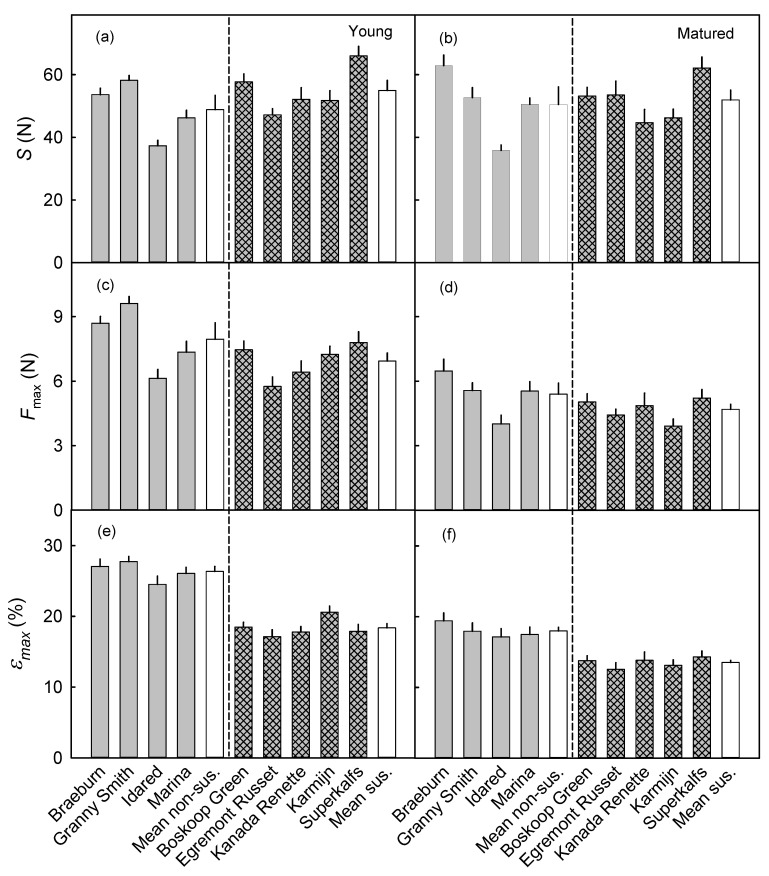
Stiffness (*S*; **a**,**b**), maximum force ($F_{max}$; **c**,**d**), and maximum strain ($\varepsilon_{max}$; **e**,**f**) of exocarp segments (ES) of 0.5 mm standard thickness excised from young (49–56 days after full bloom) (**a**,**c**,**e**) and mature (**b**,**d**,**f**) fruit of selected apple cultivars belonging to russet non-susceptible (**left**) and russet-susceptible (**right**) groups (separated by a vertical dashed line). Mean non-sus. and Mean sus. represent the mean of russet non-susceptible and russet susceptible groups, respectively. The numbers of replicates were 20–29 (young) and 15–24 (mature).

There was little difference in the *S* of the skins of russet non-susceptible and susceptible cultivars in young or mature fruit. The mean *S* within each of the two groups of cultivars were about the same at both stages of development (Figure 7a,b).

The skin of the non-susceptible cultivars “Braeburn” and “BGranny Smith” had a higher $F_{max}$ than that of susceptible cultivars in young fruit (Figure 7c). However, Idared, a non-susceptible cultivar, was an exception. This cultivar had a lower $F_{max}$, although it was a member of the non-susceptible cultivars. The mean $F_{max}$ of non-susceptible cultivars was slightly higher than that of the susceptible cultivars. At maturity, the $F_{max}$ of both groups of cultivars was similar (Figure 7d).

The $\varepsilon_{max}$ differed consistently between the two groups of cultivars. The $\varepsilon_{max}$ was higher for the skins of the non-susceptible than for those of the susceptible cultivars. This difference was larger for young than for mature fruit (Figure 7e,f).

## 3. Discussion

Major findings of our study were: (1) Compared to the non-susceptible cultivars, the epidermal and hypodermal cells in the susceptible cultivars were larger and more variable in size, particularly during early fruit development. (2) The skins of the susceptible cultivars showed lower fracture strains than those of the non-susceptible cultivars, particularly in the susceptible young stages.

### 3.1. In Early Fruit Development, the Epi- and Hypodermal Cells Were Larger and Their Sizes Were More Variable in Susceptible than in Non-Susceptible Cultivars

The differences in the size distributions of the EC and HC were generally consistent between the two groups of cultivars. Egremont Russet was an exception. Based on cell sizes, this cultivar behaved like a russet non-susceptible cultivar, yet it is highly russet susceptible. The finding of broader cell-size distributions is consistent with some earlier studies [8,23,24]. Their microscope images and drawings of cross-sections of fruit skins of russet-susceptible apple cultivars reveal an irregular structure of the epidermis. However, quantitative data is lacking for this property. Cell sizes, cell shape, and the arrangement of cells in layers were more variable in susceptible than in non-susceptible cultivars. Eccher [25] suggested that the irregularity of epidermal and hypodermal cells increased the incidence of cuticular cracking. More recent data obtained in studies of the effect of plant growth regulators also revealed a negative correlation between cuticular cracking/russeting and epidermal cell density [17,26]. A higher density and smaller size of epidermal cells as a result of the application of plant growth regulators was associated with reduced russet susceptibility.

The mechanistic basis for the production of larger and more variable-sized cells in skins (epidermis and hypodermis) of susceptible cultivars is unknown. There are several possible explanations. Variable cell sizes may result from one or several of the following: 

First, endoduplication may be a factor. Endoduplication occurs in cells of peel and cortex of various fruit crops including pear [27], apricot [28,29], tomato [30,31,32]. It results in an increase in volume in those cells that are of higher ploidy [27,28,29,30,31,32,33,34]. To our knowledge there are no reports of endoduplication in cells of apple fruit skin.

Second, differences in osmotic potential between individual cells may result in differential expansion. Cells that have a more-negative osmotic potential are expected to expand more than those having a less-negative osmotic potential. Surprisingly, large differences in cell osmotic potential have been observed in sweet cherry fruit, even over quite short distances [35]. In sweet cherry, considerable size variability exists between cells in the fruit skin. To our knowledge, there are no reports for apples. 

Finally, exogenous factors, including environmental conditions, also affect cell sizes. However, such effects are unlikely to vary over short distances within the skin, and hence are unlikely to be factors here.

### 3.2. Fracture Strain of the Skin of Non-Susceptible Cultivars Was Higher than That of Susceptible Cultivars 

The mechanical properties of the apple fruit skin are determined by the epidermal and hypodermal cell layers, but the cuticle’s contribution is minimal [2,22,36]. Hence, mechanical properties must be related to the characteristics of cells of the skin and to those of their cell walls. To our knowledge, there is no information on differences in cell wall chemistry between russet susceptible and non-susceptible cultivars. Our anatomical studies reveal that the russet-susceptible cultivars generally have more variable and, on average, larger cell sizes in the skin. This effect was fairly consistent between the two groups of cultivars with Egremont Russet being an exception. It is hypothesized that the fruit skin is weakened if cell size is heterogeneous and/or if the cells are larger. As the fruit surface expands during development without the division of cells, the periclinal walls of cells must increase, with these walls suffering increasing strain. If the strain in the walls of the EC and HC is to remain constant (i.e., total cell wall area is to remain constant), the expansion of the fruit’s surface requires the area of periclinal cell wall to be increased at the expense of a decrease in the area of the anticlinal cell wall. This process typically results in a change in the aspect ratios of the epidermal and hypodermal cells. This was described as early as 1937, by Bell [37], and in 1944, by Meyer [8]. Furthermore, to accommodate the increase in periclinal area, the anticlinal cell walls of neighboring cells must separate and reorient and so form part of the periclinal cell wall. Cell wall reorientation focuses cuticular strain on the areas immediately above the anticlinal cell walls [38]. The separation of anticlinal cell walls is accompanied by a weakening of the skin in the tangential plain, as was observed in our study. The cell-wall reorientation hypothesis also accounts for the characteristic pattern of cuticular microcracking in which the microcracks are aligned above the anticlinal walls of the epidermal cells [2]. This explanation is also consistent with large changes in fracture strain during the early stages of fruit development. At this stage, the RSGR is also maximal which decreases as fruit growth continues. These factors combine to render apple fruits particularly susceptible to skin failure and to russeting during the early stage of development.

In addition, the pattern of cell division in the fruit skin has been reported to be non-uniform in the russet susceptible cultivars. In particular, periclinal cell division followed by anticlinal division resulted in an irregular epidermis (with wider variations in cell size) in susceptible “Golden Delicious” apple [8]. Variation in cell size is likely to lead to stress concentrations. Cell walls of small cells are expected to be less strained and, hence, thicker than those of large cells. 

This being the case, smaller cells will be more rigid than larger ones causing concentration of tangential stresses at interfaces between islands of generally-smaller and generally-larger cells. This explanation is consistent with the observation that lenticels cause stress concentration in the surrounding skin. Lenticels represent a more rigid structure. When tangentially strained, the surrounding extensible skin has to make up for the relative lack of extension of the relatively stiff lenticel [39]. The above is also supported by observations made in *Arabidopsis* leaf lamellae, where negative correlations were reported between cell size and cell wall cellulose content [34]. The larger cells had higher ploidy levels, apparently associated with larger strains and so a "thinning" of the cell wall. Because cellulose is the primary determinant of the mechanical properties of apple skin tissues [40], preferential cell-wall thinning in the larger cells will weaken the fruit skin at these points. 

Such effects can be offset by the deposition of fresh cellulose on the morphological inner surfaces of the cell walls, resulting in gradients in elastic strain. Such gradients have been reported for epidermal cells of the sunflower hypocotyl, the barley coleoptile, the dandelion peduncle, and the subepidermal collenchyma of dandelion peduncle [41]. Little is known about these processes in general and about the apple fruit skin in particular. 

Lastly, cell turgor is known to alter the mechanical properties of a tissue. Oey et al. [42] reported decreased fracture strain of apple fruit tissues with high cell turgor. Similar results have been reported for pear [43]. If these factors are also applicable to russet formation in apple, the skin cells of the russet-susceptible cultivars should have higher turgor. However, we cannot think of a reason why this should be. 

### 3.3. Conclusions

Based on the results obtained in this study, we suggest the following sequence of events is likely to trigger russeting in the susceptible cultivars. The EC and HC of the russet-susceptible cultivars are larger and more variable in size. The greater size variability decreases the value for the skin’s $\varepsilon_{max}$. This occurs particularly during the early stages of fruit development when apple fruits are known to be more russet susceptible. The decrease in $\varepsilon_{max}$ causes skin failure and this in turn triggers periderm formation. Hence, from a breeder’s perspective, it is important to identify and eliminate new genotypes characterized by an irregular skin structure, as indexed by elevated variability in epidermal and hypodermal cell size and by increased populations of larger cells. From a producer’s perspective, the use of plant growth regulators such as GA_4 + 7_ may be helpful in eliminating heterogeneity of cell sizes and orientation.

## 4. Materials and Methods

### 4.1. Plant Materials

Apple fruit (*Malus × domestica* Borkh.) of the cultivars “Kamijn de Sonnaville” (hereafter referred to as Karmijn) and Idared grafted on “M9” rootstocks were obtained from the experimental orchard of the Leibniz University of Hanover at Ruthe, Germany (52°14’ N, 9°49’ E). Fruit of Braeburn, Granny Smith, "Marina", "Boskoop Green", Egremont Russet, Kanada Renette, and Superkalfs were sampled at the Federal Fruit Variety Office, Wurzen, Germany (51°22’ N, 12°45’ E). These nine apple cultivars represent two distinct groupings – one that is highly russet susceptible (Boskoop Green, Egremont Russet, Kanada Renette, Karmijn, and Superkalfs) and one that is highly russet non-susceptible (Braeburn, Granny Smith, Idared, and Marina). Trees were cultivated according to current regulations for integrated fruit production. In Idared and Karmijn, a developmental time course was first established by sampling fruit at one- to three-week intervals from fruit set through to maturity. Fruit maturity was determined by the starch pattern index [44,45,46]. One or two fruit per tree were harvested from a total of 30 trees. Fruits were held for up to two days at 2 °C. For the group comparison, fruit were sampled at three stages of development (28–35 and 49–56 DAFB and at maturity). Fruit were harvested randomly from three to five trees per cultivar. For the skin morphology study, the young stage represented fruit sampled between 28 and 35 DAFB. Whole fruit or skin samples were cut and preserved in Karnovsky’s fixative [47]. Samples were held at 2 °C until used (max. 12 months). For tensile testing, the young stage represented fruit sampled between 49 and 56 DAFB because the minimum fruit size was not achieved until about 49 DAFB, so fruit of this slightly later stage was used to measure the mechanical properties. 

### 4.2. Fruit Surface Area Growth

Fruit height and two orthogonal equatorial diameters were measured using digital calipers. Assuming sphericity, fruit surface area was calculated from the mean of the height and the two diameters. SGR was calculated as the slope of a plot of surface area vs. time using the first derivative of a sigmoidal regression model. RSGR at any time was equal to the SGR, divided by the surface area at that time. 

### 4.3. Preparation of Microscopic Images

Fruit or skin samples were removed from the fixative, rinsed in deionized water, and fruit skin cross-sections cut by hand with a sharp razor blade from equatorial region of fruit. Sections were stained for cellulose in 0.1% aqueous calcofluor white (fluorescent brightener 28; Sigma-Aldrich, Munich, Germany) for 3 to 5 min, rinsed in deionized water, placed on a microscope slide and transferred to the stage of a fluorescence microscope (BX-60; Olympus Europa, Hamburg, Germany). Specimens were observed under incident UV light (filter U-MWU 330–385 nm excitation, ≥420 nm emission). Calibrated images were prepared at 10× magnification using a digital camera (DP71, Olympus) and the Cell^P^ software (Olympus). Earlier experiments [22] established that both EC and HC are approximately isodiametric in the tangential plane. Therefore, all subsequent observations and measurements of cells were made only in latitudinal cut (across pedicel-calyx fruit axis) surfaces of the sections. No measurements were made of the cuticle, as already discussed. 

### 4.4. Epidermal and Hypodermal Cell Lengths, Heights, Tangential Areas, and Number of Cells Per Unit Area 

Cell length (tangential) and height (radial) were determined in the epidermis (1st cell layer) and in the hypodermis (2nd to 5th cell layers) using image analysis. Cells were selected for measurement by overlaying a 120 μm long transect line (5 to 12 DAFB) or 210 μm (29 to 135 DAFB) over the epidermis or hypodermis. The tangential lengths and radial heights of all cells intersecting the transect line were measured. The average number of EC and HC measured per image were 15 EC and 30 HC for the 120 μm line (5 to 12 DAFB) and 12 EC and 29 HC for the 210 μm line (29 to 135 DAFB). The total number of images analyzed per cultivar was 7 from each of 7 different fruit. 

The radial aspect ratio of the cells was calculated by dividing cell height by cell length. Values for tangential and total cell surface area of EC and HC were calculated from cell height and cell length assuming (1) that cells were isodiametric in the tangential plane (i.e., cell length = cell width) of the skin (2) that cell shape represents a rectangular prisms. Isodiametric shape of EC and HC was confirmed in an earlier experiment [22]. Cell tangential area refers to the area of the tangential face of a cell (i.e., in the plane of the fruit surface). Cell total surface area refers to the sum of all faces of the cell (both tangential faces and all radial faces).

The numbers of EC and HC per unit fruit surface area was calculated by dividing unit area by the square of the mean lengths of the EC and the HC, respectively. The numbers of EC and of HC per fruit were calculated by multiplying the appropriate cell number per unit area by the fruit surface area.

### 4.5. Uniaxial Tensile Test

Mechanical tests of ES strips were carried out following the method established earlier [22]. Briefly, ES were excised in the equatorial plane of the fruit using a custom biconcave (dumbbell shaped) punch with a “waist” width of 4.25 mm and a length of 30 to 40 mm. Excised ES were carved by hand using a sharp razor blade to thicknesses ranging from 0.5 to 1.8 mm. Calibrated images of cross-sections of the ES were prepared subsequently under a binocular microscope (MZ10 F; Leica Microsystems, Wetzlar, Germany). The thicknesses of ES were determined by image analysis. The ES were then mounted between the clamps of a material testing machine (Z 0.5 and KAP-TC 50-N force transducer; Zwick/Roell, Ulm, Germany). The free length of the specimen between the two clamps was 16 mm and the position of the waist was arranged to be in the center. Uniaxial tensile tests were carried out on the ES at a constant strain rate of 3 mm min^–1^ until failure. Tests were carried out at 22 °C temperature and 50% relative humidity (RH). Maximum force ($F_{max}$ in Newtons) at failure, maximum strain ($\;\varepsilon_{max}$ in mm mm^-1^) at failure, and stiffness (*S* in Newtons) were determined on an individual ES basis. These data were subsequently plotted as a function of thickness of the respective ES for each cultivar and sampling date. Linear regression lines were fitted through plots of $F_{max}$ or $\varepsilon_{max}$ or *S* vs. ES thickness. From the resulting regression equations values of $F_{max}$, $\varepsilon_{max}$, and *S* for each ES were calculated for a standard ES thickness of 0.5 mm. This thickness approximately matches the thickness of the fruit skin (cuticle + epidermis + hypodermis). Tests were carried out with 15 to 35 replications, where each replicate represented a different fruit.

### 4.6. Data Analyses and Presentation

Data are presented as means ± SE, as boxplots using individual data points, or as quartiles of the normal distribution plots. In the plots of mean ± SE, the absence of error bars indicates that bars were smaller than the data symbols. In boxplots, the solid and dotted horizontal lines inside the box represent the mean and median, the lower and upper ends of the box the 25 and 75 quartiles and the whiskers the 10 and 90 percentiles, respectively. Data symbols beyond the whiskers represent outliers. Data were subjected to analysis of variance, analysis of frequency distribution and regression analysis using the statistical software package SAS (version 9.1.3; SAS Institute Inc., Cary, NC, USA).

## Figures and Tables

**Table 1:** Fruit surface area and epidermal and hypodermal cell number per fruit and per unit fruit surface area of young (28–35 days after full bloom) and mature fruit of selected russet non-susceptible and russet-susceptible apple cultivars. Results for ‘Idared’ and ‘Karmijn’ were taken from Figure 2. Data in the table represent means ± SE. The number of replicates was seven.

Cultivars	Fruit Surface Area (cm^2^)		Cell No. (×10^6^ cm^−2^)				Cell No. (×10^6^ Per Fruit)			
			Epidermis		Hypodermis		Epidermis		Hypodermis	
	Young	Mature	Young	Mature	Young	Mature	Young	Mature	Young	Mature
**Russet Non-Susceptible**										
Braeburn	12.0 ± 0.4	189.5 ± 5.2	0.64 ± 0.05	0.24 ± 0.03	0.89 ± 0.03	0.33 ± 0.03	7.7 ± 0.6	45.6 ± 6.5	10.7 ± 0.4	62.0 ± 6.2
Granny Smith	9.6 ± 0.2	182.3 ± 4.4	0.70 ± 0.07	0.21 ± 0.02	1.20 ± 0.09	0.46 ± 0.04	6.8 ± 0.6	38.9 ± 3.9	11.5 ± 0.9	84.4 ± 7.0
Idared	18.5 ± 0.5	187.1 ± 3.2	0.80 ± 0.08	0.28 ± 0.02	1.07 ± 0.07	0.59 ± 0.05	14.3 ± 1.4	49.8 ± 2.7	19.2 ± 1.3	104.8 ± 8.9
Marina	13.3 ± 0.4	188.1 ± 5.8	0.72 ± 0.04	0.32 ± 0.02	1.11 ± 0.04	0.47 ± 0.03	9.7 ± 0.5	61.1 ± 4.6	14.8 ± 0.6	89.2 ± 5.0
Mean	13.4 ± 0.4b *^Z^*	186.8 ± 2.2 a	0.72 ± 0.03 a	0.26 ± 0.01 a	1.07 ± 0.04 a	0.46 ± 0.03 a	9.6 ± 0.7 a	48.8 ± 2.7 a	14.0 ± 0.8 a	85.1 ± 4.4 a
**Russet Susceptible**										
Boskoop Green	16.2 ± 0.5	180.7 ± 6.4	0.44 ± 0.03	0.20 ± 0.01	0.73 ± 0.04	0.27 ± 0.03	7.1 ± 0.5	35.6 ± 1.7	11.8 ± 0.7	48.8 ± 4.6
Egremont Russet	13.6 ± 0.5	111.7 ± 4.1	0.67 ± 0.05	0.31 ± 0.03	1.01 ± 0.06	0.57 ± 0.05	9.1 ± 0.7	34.2 ± 3.2	13.8 ± 0.8	64.0 ± 6.0
Kanada Renette	19.4 ± 0.3	130.4 ± 4.2	0.44 ± 0.04	0.22 ± 0.03	0.89 ± 0.07	0.38 ± 0.03	8.6 ± 0.7	28.4 ± 3.4	17.3 ± 1.4	49.9 ± 3.9
Karmijn	20.3 ± 0.6	146.0 ± 2.6	0.49 ± 0.06	0.28 ± 0.04	0.76 ± 0.03	0.35 ± 0.05	10.0 ± 1.1	41.3 ± 5.3	15.5 ± 0.6	52.7 ± 7.7
Superkalfs	15.4 ± 0.4	171.7 ± 4.4	0.49 ± 0.04	0.24 ± 0.02	0.59 ± 0.05	0.20 ± 0.02	7.5 ± 0.6	41.6 ± 3.1	9.1 ± 0.7	35.1 ± 3.5
Mean	17.3 ± 0.3 a	143.0 ± 3.3 b	0.51 ± 0.02 b	0.25 ± 0.01 a	0.80 ± 0.03 b	0.36 ± 0.03 b	8.5 ± 0.4 a	36.2 ± 1.7 b	13.5 ± 0.6 a	50.1 ± 2.8 b

*^Z^* Means of groups of cultivars within a column followed by the same letter are not significantly different, Tukey studentized range test at *p* ≤ 0.05.

**Table 2:** Epidermal and hypodermal cell parameters of young (28–35 days after full bloom) and mature fruit of selected russet non-susceptible and russet-susceptible apple cultivars. Results for ‘Idared’ and ‘Karmijn’ were taken from the Figure 3. Data in the table represent means ± SE. The numbers of cells measured were 68–145 (epidermis) and 172–331 (hypodermis) cells in young and 57–97 (epidermis) and 128–205 (hypodermis) cells in mature fruit. Cells were measured in seven fruit per cultivar.

Cultivars	Cell Height (μm)				Cell Length (μm)				Tangential Area (μm^2^)				Total Surface Area (μm^2^)			
	Epidermis		Hypodermis		Epidermis		Hypodermis		Epidermis		Hypodermis		Epidermis		Hypodermis	
	Young	Mature	Young	Mature	Young	Mature	Young	Mature	Young	Mature	Young	Mature	Young	Mature	Young	Mature
**Russet Non-Susceptible**																
Braeburn	16.2 ± 0.2	11.7 ± 0.3	12.3 ± 0.2	17.6 ± 0.5	12.6 ± 0.3	21.6 ± 0.8	21.2 ± 0.4	35.6 ± 1.2	171 ± 9	503 ± 36	502 ± 22	1535 ± 126	1167 ± 43	2018 ± 113	2084 ± 78	5914 ± 460
Granny Smith	18.1 ± 0.3	15.7 ± 0.3	13.0 ± 0.2	15.2 ± 0.3	12.0 ± 0.3	22.0 ± 0.7	18.4 ± 0.4	29.9 ± 0.9	155 ± 8	522 ± 33	381 ± 17	1056 ± 69	1182 ± 44	2446 ± 119	1740 ± 61	4058 ± 242
Idared	13.2 ± 0.2	11.3 ± 0.3	11.5 ± 0.2	13.6 ± 0.3	11.3 ± 0.4	19.2 ± 0.6	19.4 ± 0.5	26.7 ± 0.8	140 ± 9	393 ± 25	426 ± 21	831 ± 51	886 ± 41	1667 ± 88	1773 ± 73	3218 ± 177
Marina	14.7 ± 0.2	11.0 ± 0.2	12.5 ± 0.2	12.9 ± 0.3	11.8 ± 0.4	17.7 ± 0.5	19.0 ± 0.4	29.2 ± 0.8	152 ± 9	329 ± 17	400 ± 17	964 ± 56	998 ± 42	1451 ± 63	1777 ± 63	3517 ± 181
Mean	15.7 ± 0.1 b *^Z^*	12.5 ± 0.2 a	12.3 ± 0.1 b	14.8 ± 0.2 a	12.0 ± 0.2 b	20.1 ± 0.3 a	19.6 ± 0.2 b	30.3 ± 0.5 b	155 ± 4 b	438 ± 15 a	429 ± 10 b	1091 ± 41 b	1070 ± 22 b	1904 ± 54 a	1852 ± 35 b	4158 ± 146 b
**Russet Susceptible**																
Boskoop Green	20.3 ± 0.3	12.6 ± 0.2	16.0 ± 0.2	15.3 ± 0.4	15.2 ± 0.4	22.9 ± 0.7	23.5 ± 0.6	38.9 ± 1.4	250 ± 14	562 ± 34	627 ± 31	1805 ± 137	1745 ± 71	2278 ± 105	2812 ± 111	6233 ± 430
Egremont Russet	15.7 ± 0.2	11.4 ± 0.2	10.9 ± 0.2	12.2 ± 0.2	12.3 ± 0.4	18.6 ± 0.6	20.0 ± 0.5	26.9 ± 0.9	162 ± 10	377 ± 25	451 ± 22	851 ± 71	1100 ± 44	1611 ± 80	1805 ± 73	3097 ± 217
Kanada Renette	17.6 ± 0.4	12.4 ± 0.2	12.0 ± 0.2	12.1 ± 0.2	15.1 ± 0.4	22.1 ± 0.9	21.4 ± 0.5	32.5 ± 1.2	242 ± 14	540 ± 52	504 ± 25	1253 ± 108	1555 ± 66	2185 ± 153	2052 ± 81	4163 ± 307
Karmijn	18.3 ± 0.3	12.7 ± 0.3	11.5 ± 0.2	15.8 ± 0.5	14.6 ± 0.5	19.4 ± 0.6	23.0 ± 0.6	34.6 ± 1.1	228 ± 13	408 ± 25	599 ± 32	1429 ± 101	1521 ± 61	1810 ± 87	2308 ± 107	5376 ± 372
Superkalfs	22.6 ± 0.4	12.6 ± 0.2	17.3 ± 0.3	19.3 ± 0.7	14.4 ± 0.5	20.8 ± 0.7	26.2 ± 0.6	45.1 ± 1.5	227 ± 14	485 ± 39	768 ± 37	2380 ± 156	1769 ± 78	2039 ± 121	3417 ± 138	8694 ± 568
Mean	19.0 ± 0.2 a	12.3 ± 0.1 a	13.6 ± 0.1 a	15.0 ± 0.2 a	14.3 ± 0.2 a	20.7 ± 0.3 a	22.8 ± 0.3 a	35.5 ± 0.6 a	221 ± 6 a	469 ± 16 a	591 ± 14 a	1534 ± 55 a	1540 ± 32 a	1968 ± 50 a	2490 ± 50 a	5505 ± 190 a

*^Z^* Means of the groups of cultivars within a column followed by the same letter are not significantly different, Tukey studentized range test at *p* ≤ 0.05.
